# Discovery of a novel RORγ antagonist with skin-restricted exposure for topical treatment of mild to moderate psoriasis

**DOI:** 10.1038/s41598-021-88492-1

**Published:** 2021-04-28

**Authors:** Suxing Liu, Dong Liu, Ru Shen, Di Li, Qiyue Hu, Yinfa Yan, Jiakang Sun, Fengqi Zhang, Hong Wan, Ping Dong, Jun Feng, Rumin Zhang, Jing Li, Lianshan Zhang, Weikang Tao

**Affiliations:** 1 Eternity Bioscience Inc., 6 Cedarbrook Drive, Cranbury, NJ 08512 USA; 2 Shanghai Hengrui Pharmaceutical Co. Ltd., 279 Wenjing Road, Shanghai, 200245 China

**Keywords:** Drug discovery, Immunology, Immunological disorders, Skin diseases

## Abstract

Clinical success of IL-17/IL-23 pathway biologics for the treatment of moderate to severe psoriasis suggests that targeting RORγt, a master regulator for the proliferation and function of Th17 cells, could be an effective alternative. However, oral RORγ antagonists (VTP43742, TAK828) with high systemic exposure showed toxicity in phase I/II clinical trials and terminated development. To alleviate the potential safety concerns, identifying compounds with skin-restricted exposure amenable for topical use is of great interest. Systematic structure activity relationship study and multi-parameter optimization led to the discovery of a novel RORγ antagonist (SHR168442) with desired properties for a topical drug. It suppressed the transcription of IL-17 gene, leading to reduction of IL-17 cytokine secretion. It showed high exposure in skin, but low in plasma. Topical application of SHR168442 in Vaseline exhibited excellent efficacy in the imiquimod-induced and IL-23-induced psoriasis-like skin inflammation mouse models and correlated with the reduction of Th17 pathway cytokines, IL-6, TNFα and IL-17A. This work demonstrated restricted skin exposure of RORγ antagonist may provide a new topical treatment option as targeted therapeutics for mild to moderate psoriasis patients and may be suitable for the treatment of any other inflammatory disorders that are accessible locally.

## Introduction

Psoriasis is a chronic immune-mediated skin disorder characterized by scaly indurated erythema. The prevalence of psoriasis is estimated at approximately 100 million people worldwide^1^. Several meta-analyses reviewed IL-23/IL-17 pathway biologics (Guselkumab, risankizumab, ustekinumab, ixekizumab, secukinumab and brodalumab) that showed superior clinical efficacy in the treatment of moderate to severe psoriasis^2–7^, suggesting that IL-23/IL-17 axis is a major immune pathway underlying the disease pathophysiology.

The levels of IL-23 and IL-17 are elevated in psoriatic skin compared with non-lesional skin, positively correlated with the development of psoriasis^8^. Overactive Th17 cells cause skin epidermal proliferation and psoriasis. Activation of the IL-23 receptor promotes and stabilizes the generation of Th17 cells, and forms part of the positive feedback loop^9^. RORγt is a master transcription factor that activates the transcription of IL-23 receptor gene in undifferentiated CD4 T helper cells. At the same time, RORγt activates the gene transcription of pro-inflammatory cytokines such as IL-17A, IL-17F, IL-21, and IL-22, and enhances the inflammatory process. Clinical success of IL-23/IL-17 pathway biologics suggests that targeting RORγt antagonists could be an effective alternative therapy for psoriasis.

Conditions from approximately 75% of psoriasis patients are considered as mild to moderate. This group of patients can benefit from topical treatment without systemic exposure. Biologics is not suitable for topical route delivery due to the large size and poor permeability into skin. Currently, topical corticosteroids and vitamin D analogues are applied to treat patients with mild to moderate psoriasis. However, these treatments not only have limited efficacy, but also cause significant side effects and patients must avoid long-term use.

It was reported that oral RORγ antagonists (VTP43742, TAK828) with high systemic exposure showed toxicity in phase I/II clinical trials and terminated development^10,11^. The skin-restricted exposure of RORγ antagonists may alleviate the safety risk due to systemic exposure while still maintaining similar efficacy as biologics in IL-23/IL-17 pathway. Our strategy is to design RORγ antagonists that are suitable for topical therapy in psoriasis. Here we report the discovery of a novel RORγ antagonist, SHR168442, that has skin-restricted exposure across species with excellent cellular on-target effects. Furthermore, SHR168442 exhibited excellent topical efficacy in two preclinical animal models for psoriasis with a desirable PK profile and the suppression of the signature cytokines. This novel RORγ antagonist may provide a new option as topical targeted therapeutics for mild to moderate psoriasis patients.

## Results

### Identification of a novel chemical scaffold with RORγ antagonistic activity

**Figure 1:**
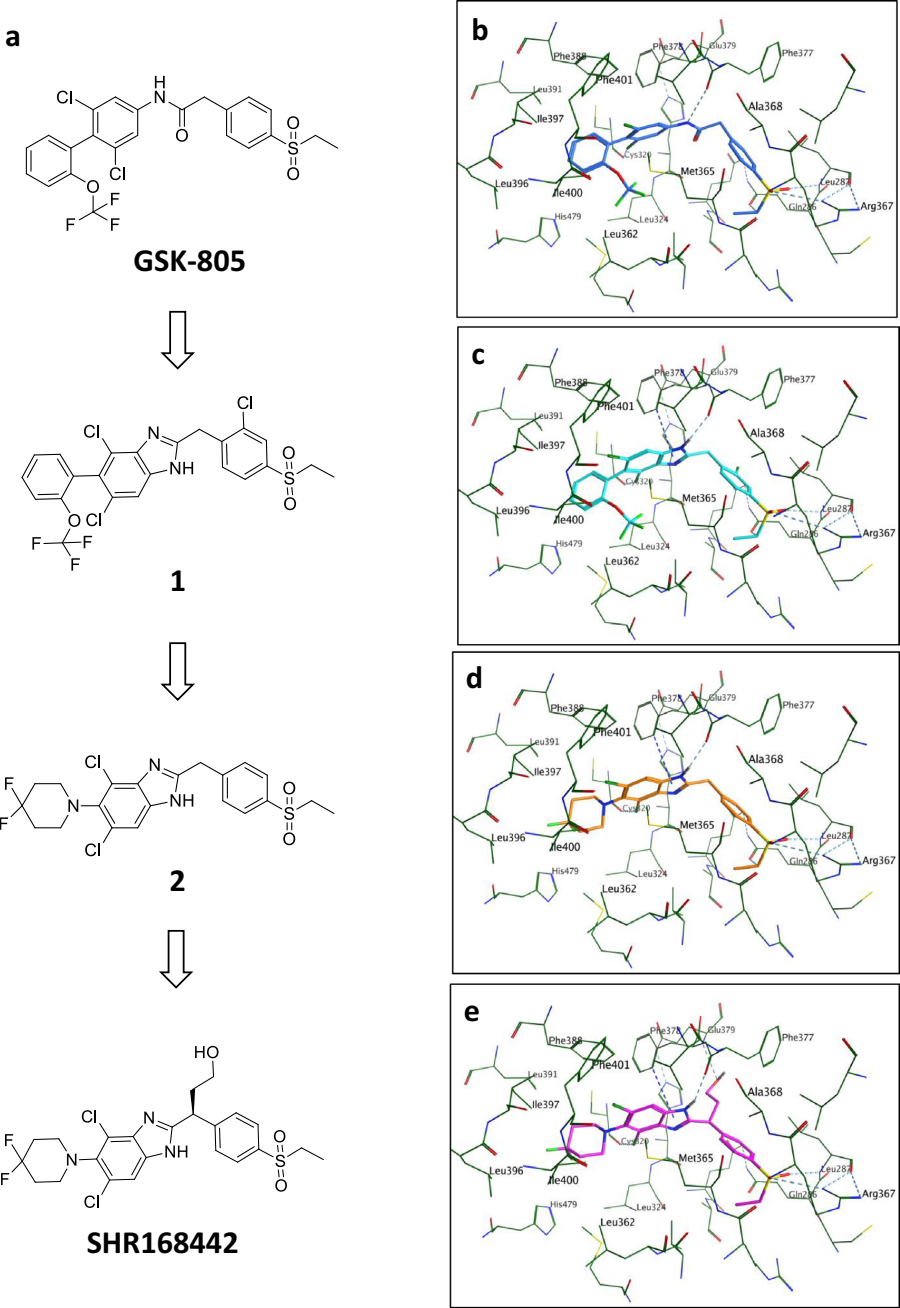
Identification of a novel series of benzimidazole as RORγ antagonists. (**a**) SAR scheme from identification of a novel chemical scaffold (Compound 1) derived from GSK805 to selected RORγ antagonists (Compound 2 and SHR168442) for skin-restricted exposure. (**b–e**) Modeled binding modes of RORγ antagonists in RORγ LBD colored in green from PDB (Accession number: 4NIE). (**b**) GSK805 colored in blue, (**c**) Compound 1 colored in cyan, (**d**) Compound 2 colored in orange, and (**e**) SHR168442 colored in magenta.

GSK reported a series of potent RORγ antagonists that are orally bioavailable, such as GSK805^12^. Based on the modeled pose of GSK805, we designed a new chemical scaffold with a benzimidazole core to mimic the amide of GSK805 while keeping the key interactions with the RORγ ligand-binding domain (LBD) such as hydrogen bonds with Cys 320, Phe 377, Gln286, Leu287 and Arg 367 (Fig. 1a,b). A series of analogues with various substituents on the right-hand side phenyl ring were synthesized (WO/2019/213470), which led to Compound 1 with excellent in vitro potency (IC_50_ = 62 nM, Fig. 1a and Table 1). The N–H of the benzimidazole core of Compound 1 maintained the same hydrogen bonding interaction with the backbone carbonyl group of Phe377 as the amide group in GSK805 (Fig. 1b,c). Interestingly, while maintaining the key interactions, there is an additional interaction picked up by Compound 1 compared to GSK805. As shown in Fig. 1c, the phenyl group of Phe378 formed an edge to face π–π interaction with the imidazole ring of Compound 1. Further structure activity relationship (SAR) study and optimization led to discovery of the lead compound, SHR168442, (S)-3-(4,6-dichloro-5-(4,4-difluoropiperidin-1-yl)-1H-benzo[d]imidazol-2-yl)-3-(4-(ethylsulfonyl)phenyl)propan-1-ol, with excellent in vitro potency and desired properties for topical usage. All compounds were synthesized at Eternity Bioscience Inc. or Shanghai Hengrui Pharmaceutical Co Ltd. The preparation of SHR168442 is described in “Methods” section and those of other compounds are described in Supplementary Methods.

**Table 1:** The profiles of selected compounds in this work.

Compound	1	2	SHR168442
Binding of RORγ LBD, IC_50_ (nM)	62 ± 18	40 ± 27	35 ± 9
IL-17 production in human PBMC, EC_50_ (nM)	30 ± 10	46 ± 2	20 ± 6
**Liver microsomal stability, t**_**1/2**_** (min)**			
Human	> 120	18.5	2.3
Rat	> 120	34.5	8.6
Mouse	nd	7.8	6.2
**Exposure in SD Rat at 2 mg/kg (oral)**			
C_max_ (ng/mL)	446*	70	9.5
AUC (ng/mL∙h)	6979*	352	59
t_1/2_ (h)	12.8*	2.0	6.2

*LBD* ligand-binding domain, *nd* not determined.

*Dosed at 5 mg/kg.

### Characterization of newly identified RORγ antagonists by LanthaScreen TR-FRET human RORγ coactivator assay

RORγ is a member of the nuclear receptor subfamily of intracellular transcription factors, consisting of DNA binding domain, ligand binding domain, hinge domain and activation domain^13^. The activation of target gene transcription involves conformational changes in the receptor upon the ligand binding, leading to dissociation of repressor proteins, association of coactivator proteins, and assembly of pol II and other transcriptional factors.

**Figure 2:**
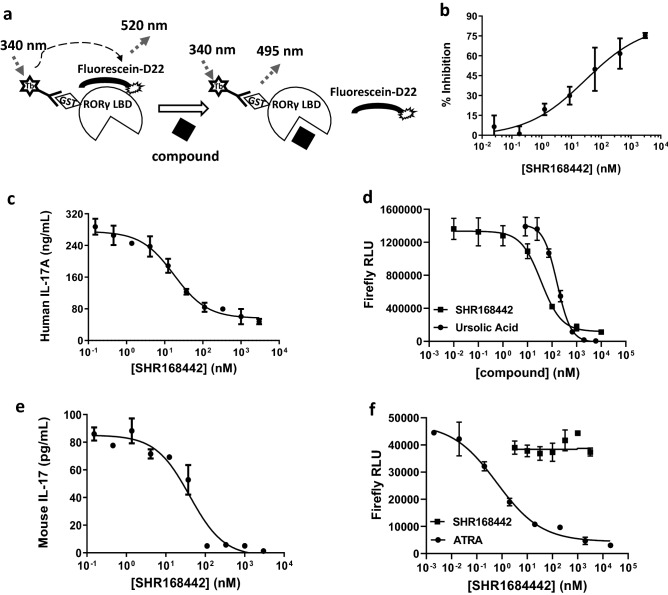
Biochemical and cellular on-target activities of SHR168442. (**a**) Principle of the LanthaScreen TR-FRET human RORγ LBD coactivator assay. The terbium (Tb)-anti-GST antibody indirectly labels the RORγ LBD by binding to the GST tag of the protein. Binding of the compound to RORγ LBD causes a conformational change that results in a decrease in the affinity of the receptor for a labeled coactivator peptide, fluorescein-D22. (**b**) Dose–response curve for SHR168442 as an antagonist in LanthaScreen TR-FRET human RORγ LBD coactivator assay. (**c-d**) SHR168442 inhibits IL-17 secretion from human PBMCs (**c**) and mouse splenocytes (**d**). (**e–f**) Effects of SHR168442 on RORγ (**e**) and RORα (**f**) luciferase reporter expression. Ursolic acid and ATRA were used as positive control in the assays, respectively. RLU = relative light units. Non-linear regression analyses were performed and IC_50_ values determined using GraphPad Prism software. Representative curves are shown. Average of the measurement and its standard deviation are shown.

In LanthaScreen TR-FRET RORγ coactivator assay, the RORγ LBD was constitutively active and the coactivator peptide (fluorescein-D22) was recruited in the absence of ligand, or compound (Fig. 2a). The binding of an antagonist to RORγ LBD causes a conformational change of the receptor, leading to a decrease in the affinity of the receptor for the coactivator peptide. The separation of the fluorescently labeled coactivator peptide from RORγ LBD from the receptor with the terbium-labeled antibody causes a decrease in the TR-FRET signal compared to those without compound. The assay was validated by detecting the ligand-independent recruitment of coactivator peptide in the presence or absence of RORγ LBD (2.5- to 3-fold assay window). Effects of the key compounds of the novel chemotype in Fig. 1, were tested in LanthaScreen TR-FRET RORγ coactivator assay. The results indicated they all showed the antagonist mode with various potency (Table 1). The dose–response curve of the lead analogue, SHR168442, is shown in Fig. 2b.

### Cellular on-target activity of selected RORγ antagonists

To confirm cellular on-target effects, we investigated the inhibition of selected RORγ antagonists on RORγ-regulated IL-17 secretion using human PBMC. Human IL-17-producing cells were readily formed following cytostim stimulation^14^. In human PBMC stimulated with cytostim for 72 h, differentiation of Th17 cells was confirmed by increased secretion of IL-17 (291 pg/mL) in cell culture supernatant compared to basal level of IL-17 (20 pg/mL IL-17 without cytostim stimulation), a 25-fold increase of IL-17 secretion. As shown in Fig. 2c, SHR168442 significantly reduced the secretion of IL-17 from human PBMCs with average IC_50_ = 20 nM. The effects of analogues on the secretion of IL-17 from human PBMCs are summarized in Table 1.

Furthermore, nuclear reporter assay to measure the RORγ transcriptional activity of SHR168442 (Fig. 2d) demonstrated the inhibition of the RORγ transcriptional activity with average IC_50_ = 15 ± 9 nM (n = 3). Ursolic acid was used as a positive control^15^. Together with the results from IL-17 production assay, it suggests that SHR168442 can suppress the transcription of IL-17 gene, leading to reduction of IL-17 cytokine secretion.

To evaluate the impact of SHR168442 on IL-17 secretion in mouse cells, the mouse Th17 cell differentiation kit from R&D Systems was used to polarize the mouse spleen cells. Differentiation of Th17 cells in mouse splenocyte for 72 h was confirmed by secretion of IL-17 in culture supernatant, 93 pg/mL in average (2.7 pg/mL without differentiation), a 34-fold increase of IL-17 secretion. As shown in Fig. 2e, SHR168442 inhibited the secretion of IL-17 in mouse splenocyte with average IC_50_ = 54 ± 12 nM (n = 3).

### High selectivity of SHR168442 for RORγ

We next assessed the selectivity of SHR168442 against another ROR family member, RORα, using nuclear reporter assay. As shown in Fig. 2f, SHR168442 had little effect on RORα reporter activity, up to 10,000 nM (n = 3), suggesting its selectivity for RORγ over RORα. ATRA (all trans-retinoic acid) was used as a positive control in the assay^16^.

To further profile any off-target activity, we counter-screened SHR168442 in SAFETYscan47 panel, compiled by DiscoverX based on the recommendation by major pharmaceutical companies^17^. SHR168442 was screened at 3 µM in duplicates. The results of SAFETYscan47 panel, shown in Supplementary Table S1, indicated that SHR168442 inhibits the targets in the panel all below 50% inhibition, suggesting that the high selectivity of SHR168442 for RORγ.

### SAR of selected RORγ antagonists for skin-restricted exposure

Compound 1 is very stable in human and rat liver microsomes (t_1/2_ > 120 min) and has high systemic exposure in SD rats when dosed orally at 5 mg/kg (AUC = 6979 ng/mL∙h) (Table 1). Nevertheless, our goal was to design and identify a molecule with low systemic exposure and other suitable properties for topical usage. Next, to make the compound metabolically less stable, we would like to replace the left-hand side benzene ring with piperidine to reduce the number of aromatic rings in the molecule while maintaining in vitro potency. As shown in Fig. 1a, the trifluoro methoxy benzenyl group of GSK805 occupies the left-hand side hydrophobic pocket formed by residues, Phe401, Phe388, Leu391, Ile397, Leu396, Ile400, His479, Leu362, Leu324 and Met365 in RORγ LBD. Modeling study suggests that the 4,4-difluoropiperidinyl group can fit into the same pocket (Fig. 1d). Compound 2 displayed similar in vitro potency (IC_50_ = 40 nM) to Compound 1 (Table 1). This supports our modeled binding mode of Compound 2, in which the piperidine moiety on left-hand side occupies similar position to the benzene ring of GSK805 and did not introduce steric clashes while the rest part of Compound 2 maintained the key interactions with the RORγ LBD (Fig. 1d). As predicted, Compound 2 has much shorter t_1/2_ in human, rat and mouse liver microsome stability assays (t_1/2_ = 7.8–34.5 min), leading to lower systemic exposure (AUC = 352 ng/mL∙h) when dosed orally at 2 mg/kg in SD rats.

Further optimization with the addition of hydroxyethyl chain to the methylene group in between the two aromatic systems of Compound 2 resulted in SHR168442, which shares the similar binding mode as Compound 2 (Fig. 1a,e). In addition, the hydroxyethyl chain picked up a direct hydrogen bonding interaction with the backbone NH group of Glu379. This substitution helped to keep in vitro potency (Table 1), improve solubility and further reduce systemic exposure (AUC = 59 ng/mL∙h). Another critical property of the compounds was the exposure in the skin. The results of the dermal PK analysis indicated that the exposure of SHR168442 in epidermis and dermis layers of BALB/c mouse was 2,460 ng/g at 3 h and 4,390 ng/g at 6 h after topical application (Table 2). The PK profiling in skin and plasma of C57BL/6 mice and SD rats were further explored and, consistently, the profiles of high exposures in skin and low in plasma were observed.

**Table 2:** Exposures of SHR168442 in Vaseline via topical route in skin and plasma of mice, rats, and miniature pigs.

Species	N	Dose (%)	Exposure in skin (ng/g)			Exposure in plasma (ng/mL)		
			3 h	6 h	24 h	3 h	6 h	24 h
BALB/c mouse	3	2	2,460	4,390	nd	blq	blq	nd
C57BL/6 mouse	3	0.5	1,457	9,707	nd	8.8	4.7	nd
	3	8	9,707	18,500	nd	7.0	55	nd
SD Rat	3	8	6,898	5,639	nd	8.7	2.4	nd
Miniature pig	2	2	232	196	581	blq	blq	blq
	2	8	5,410	5,975	7,430	blq	blq	blq

*nd* not determined, *blq* below limit of quantitation (< 6 ng/mL).

Due to the comparability of pig skin integument to human’s skin, the dermal PK of miniature pigs was determined. As shown in Table 2, similar exposures of SHR168442 in miniature pigs were observed at 3 h and 6 h, and the highest exposure was reached at 24 h after topical applications in both doses (2% and 8%). Much higher exposures appeared in 8% cream across all three time points than in 2%. Consistent with the observation with those in other species, the systemic exposures of SHR168442 in miniature pigs were all below detection level (< 6 ng/mL).

### Inhibitory effects of SHR168442 via topical routes in an imiquimod (IMQ)-induced psoriasis-like inflammation model

**Figure 3:**
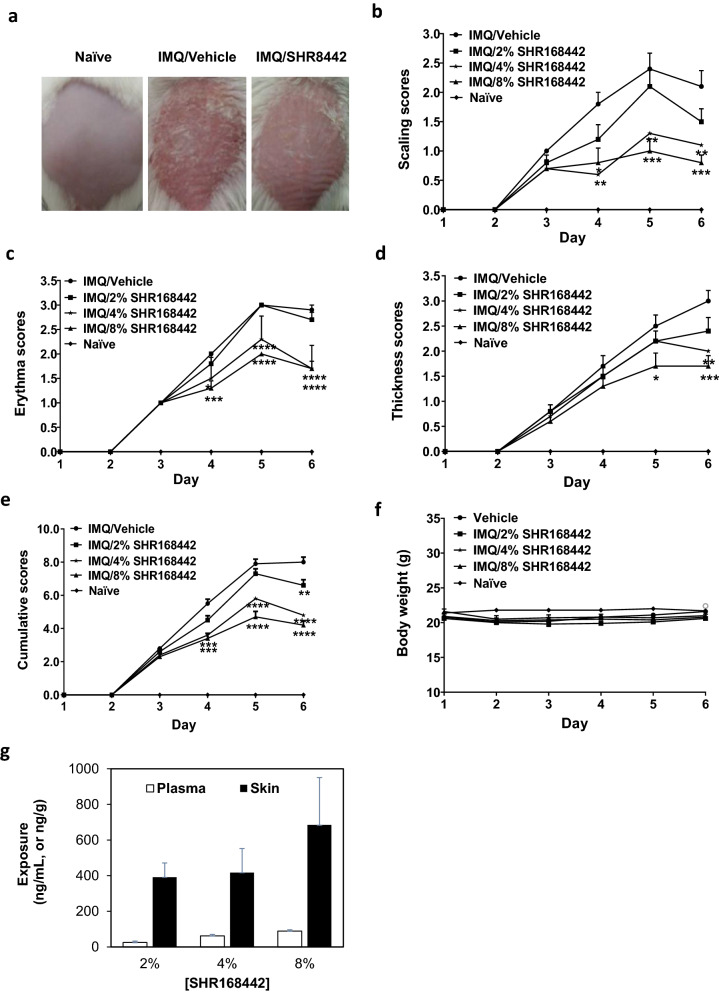
Effect of the topical treatment of SHR168442 in multiple doses (2%, 4% and 8%) in IMQ-induced psoriasis-like skin inflammation mice.(**a**) Representative photos of mice in naïve group or the groups of treated topically with IMQ and Vaseline or 8% SHR168442 at day 6.  (**b–d**) Scaling (**b**), Erythema (**c**), and thickness (**d**) of the back skin were scored daily on a scale of 0 to 4. (**e**) Cumulative scores were presented as the sum of the scores of scaling, erythema and thickness. Symbols indicated mean score ± SEM of 10 mice per group. Vehicle = Vaseline. Data were analyzed by one-way ANOVA, *p < 0.05, **p < 0.01, ***p < 0.001, ****p < 0.0001 versus vehicle. (**f**) Body weights of mice were measured daily. (**g**) Exposures of SHR168442 in Vaseline are high in diseased skin and low in plasma in IMQ-induced psoriasis-like skin inflammation mice. The exposure of SHR168442 in dorsal skin (ng/g, black bar) and in plasma (ng/mL, open bar) on day 8 of the study are shown. Symbols indicated mean ± SEM of 4 mice per group.

As shown in Fig. 3a, the topical application of 5% IMQ cream on hair-free back of BALB/c mice resulted in the development of psoriasis-like lesions within 6 days, consistent with the report^18^. At two days after the IMQ application, the back skin of the mice started to display significant signs characteristic of psoriasis, such as erythema, scaling, and thickening, which continually increased in severity up to the end of the study (Fig. 3b,f). The mice treated with SHR168442 exhibited clear dose-dependent reduction based on cumulative scores (Fig. 3e). As shown in Fig. 3b, the significant reduction of the score of scaling (< 1.0) by the treatment of 8% SHR168442 was observed from days 4 to 6 at days 5 and 6 as compared with vehicle control (*p* < 0.001). Similar trend was observed with thickness scores at day 6 (Fig. 3d). In addition, the scores of erythema were significantly reduced at day 6 of treatment of 4% and 8% of SHR168442 as compared with vehicle control (Fig. 3c). There was no significant body weight loss in all treatment groups (Fig. 3f). At the end of the study, the mouse plasma and diseased back skin were collected 3 h after topical application for PK analysis. As shown in Fig. 3g, the exposures of SHR168442 are high in skin and low in plasma across all treatments. Taken together, the results indicated that SHR168442 in Vaseline was efficacious in a dose-dependent manner and achieved favorable skin-restricted exposure by topical application in an IMQ-induced psoriasis-like inflammation model.

### Inhibitory effects of SHR168442 via topical routes in IL-23-induced psoriasis-like inflammation mouse model

**Figure 4:**
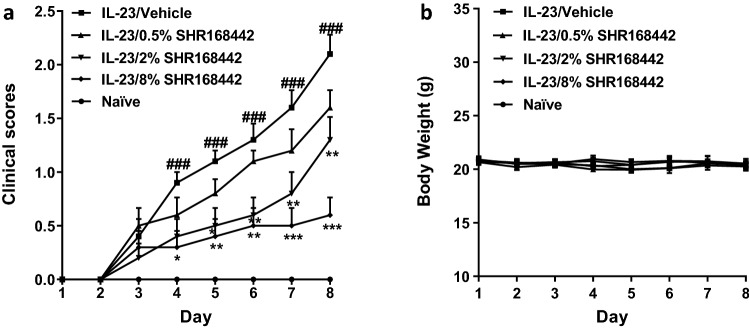
Effect of the treatment of SHR168442 in Vaseline in IL-23-induced psoriasis-like skin inflammation mice. (**a**) Clinical scores of the dorsal skin were measured daily on a scale of 0 to 4. Symbols indicated mean ± SEM of 10 mice per group. Vehicle = Vaseline. Data were analyzed by one-way ANOVA, ^###^ *p* < 0.001 versus Naïve; *p < 0.05, **p < 0.01, ***p < 0.001 versus vehicle. (**b**) Body weights of mice were measured daily.

The in vivo efficacy of SHR168442 was further investigated in IL-23-induced psoriasis-like mouse model^19^. As shown in Fig. 4a, there is a statistically significant difference in clinical scores from day 4 to the end of study between naïve group and IL-23/vehicle group, suggesting IL-23 induced psoriasis-like inflammation well. Furthermore, SHR168442 can reduce clinical scores in a dose-dependent manner. The severity of the psoriasis-like inflammation of the mice treated with 8% SHR168442 exhibited the most significant reduction of clinical scores starting on day 4 and maintained low clinical scores to the end of the study (day 8). There was no significant body weight loss in all treatment groups (Fig. 4b).

IL-23-injected mice had thicker skin when compared with saline-injected skin^19^. The skin thickness of the mice in the vehicle group (0.564 mm) was increased significantly as compared with the naïve group (0.324 mm, *p* < 0.001), confirming IL-23 induced psoriasis-like inflammation. Consistent with clinical scores, changes of skin thickness by topical treatment of SHR168442 show dose-dependent decrease (Table 3) and displayed the most improvement at 8%, close to the skin thickness of naïve group (97% reduction, *p* < 0.001). All treatments are well tolerated with little changes in body weight (Fig. 4b).

**Table 3:** Effects of SHR168442 in Vaseline via topical route in skin thickness of mice induced by IL-23 (Mean ± SEM, n = 10).

Group	Thickness of the back skin (mm)
Naïve	0.324 ± 0.014
IL-23/vehicle	0.564 ± 0.029^###^
IL-23/0.5% SHR168442	0.452 ± 0.020*
IL-23/2% SHR168442	0.436 ± 0.019**
IL-23/8% SHR168442	0.330 ± 0.015***

Data were analyzed by one-way ANOVA.

^###^*p* < 0.001 versus naïve group.

*p < 0.05, **p < 0.01, ***p < 0.001 versus IL-23/vehicle group.

After intradermal injection of IL-23, cytokines secreted by various cells enter the peripheral blood to form a hyperimmune physiological environment, which is an important factor in the development of mouse psoriasis-like inflammation model. As shown in Table 4, the concentrations of IL-6, TNFα and IL-17A in the serum of mice at the peak of disease onset were significantly higher than those in the naïve mouse group (*p* < 0.001). The levels of IL-6, TNFα and IL-17 were significantly reduced as compared to the vehicle group, suggesting SHR168442 suppressed the production of Th17 pathway related cytokines in IL-23-induced Psoriasis-like skin inflammation mice.

**Table 4:** Effects of SHR168442 in Vaseline on production of multiple cytokines via topical route (Mean ± SEM, n = 10).

Group	IL-6 (fg/mL)	TNFα (fg/mL)	IL17 (fg/mL)
Naïve	120 ± 14	4131 ± 532	152 ± 22
IL-23/vehicle	300 ± 29^###^	12,138 ± 1699^###^	295 ± 34^###^
IL-23/0.5% SHR168442	124 ± 32***	5495 ± 837**	190 ± 28*
IL-23/2% SHR168442	112 ± 43***	4284 ± 804***	172 ± 38*
IL-23/8% SHR168442	123 ± 37***	3380 ± 491***	185 ± 35*

Data were analyzed by one-way ANOVA.

^###^*p* < 0.001 versus naïve group.

*p < 0.05, **p < 0.01, ***p < 0.001 versus IL-23/vehicle group.

## Discussion

Oral RORγ antagonist VTP43742 with high systemic exposure showed a clear signal of efficacy in psoriatic patients over a four-week period in phase 2a clinical trial, providing the proof of concept of RORγ antagonist for treatment of psoriasis^10^. However, elevations of reversible transaminase were observed and prompted the termination of development. Similarly, the phase I trial of oral RORγ antagonist TAK-828F was discontinued based on results of preclinical toxicology and clinical teratogenicity studies^11^. Systemic treatment of RORγ antagonists for inflammation may cast a safety risk due to cross reactivity with RORγ1 and RORγt, and potential adverse events in developing T-cell lymphoma. RORγt is a differentially spliced isoform of RORγ1, 19 amino acids shorter at N-terminus^9,13^. The compounds tested biochemically using LBD of the receptor will result in pan-RORγ antagonists. RORγt is expressed predominantly in immune cells, including Th17, Tc17, γδ T cells and regulatory T cells^9,20–23^. Whereas RORγ1 is expressed in a variety of organ systems as well as in Th17 cells^24^. In addition, 50% of embryonic RORγ deficient mice develop T-cell lymphoma^25^. Lymphoma was also observed in adult RORγ knockout mice with immune systems intact^26^.

Topical treatment of RORγ antagonists could alleviate this safety risk while still maintaining similar efficacy as biologics in IL-23/IL17 pathway. It was reported that RORγ antagonist GSK2981278 with topical application failed in phase 1 trial for psoriasis^27^. GSK2981278 ointment was applied in 0.03%, 0.1%, 0.8% and 4%. Infiltrate thickness was not changed across all doses. Biomarker results suggest the target was not engaged. Although GSK2981278 is a highly potent and selective antagonist of RORγ in vitro, GSK2981278 exhibited limited efficacy in reduction of epithermal thickness (23% reduction vs placebo + IMQ control at 1% GSK2981278 ointment) in IMQ-induced mouse psoriasis-like inflammation model^28^. While low systemic exposure of GSK2981278 in mouse serum was reported, the exposure in the skin tissue was not disclosed^28^. The lack of efficacy in phase 1 trial may be due to insufficient drug exposure at the target site^27^.

Our strategy is to design and optimize the efficacy and safety profiles of RORγ antagonists in prolonged action at the skin while being rapidly eliminated from the systemic circulation, which are suitable for topical therapy in psoriasis. In this work, a novel series of benzimidazole with RORγ antagonistic activity was identified through core replacement of GSK805 (WO/2019/213470). Based on the information from the molecular modeling and activity-guided structural optimization (Fig. 1), we aimed to make compounds metabolic unstable and minimize the systemic exposure while keeping in vitro potency and high exposure in skin (Table 1). A novel RORγ antagonist (SHR168442) was identified with excellent on-target cellular activities and high selectivity for RORγ (Fig. 2 and Table 1). More importantly, it exhibited favorable skin-restricted exposures in vivo across species (Table 2).

In the dermis of the psoriasis skin lesion, the number of T cells was dramatically increased as compared with normal skin^8^. For topical treatment, the ability for a small molecule to penetrate the stratum corneum and reach the dermis is critical. The dermal PK sampling of a topical drug was carefully assessed by the skin stripping technique^29^. The dermal delivery and systemic exposure of SHR168442 were first carried out in two mouse strains (BALB/c and C57BL/6) used for the efficacy studies. The dermal PK results of SHR168442 in BALB/c and C57BL/6 mice all showed high exposures in skin and low in plasma (Table 2). Similar PK profile was observed in SD rats. Miniature pigs have been used extensively in dermal research because of comparability of their integument to human’s skin^30,31^. To better predict the dermal delivery of SHR168442 in humans, dermal PK study of SHR168442 was carried out on miniature pigs. The results confirmed high and sustained skin exposures of SHR168442 in dose-dependent manner and systemic exposures all below detection level (Table 2).

In a survey of 17,425 psoriasis patients, the most frequent symptoms experienced were scaling (94%), itching (79%), and erythema (71%)^32^. IMQ-treated mouse skin closely resembles human psoriasis lesions with respect to scaling, erythema, skin thickening^18^. Furthermore, genetic knockout of IL-23 and IL-17 receptors individually leads to a nearly complete blockade of disease, despite daily IMQ application during the entire 6-day experimental period, suggesting the lesion development is critically dependent on IL-23 and IL-17. Therefore, the inhibitory effects of SHR168442 in Vaseline were evaluated via topical delivery in IMQ-induced and IL-23-induced psoriasis-like inflammation models. The results (Figs. 3, 4 and Tables 3, 4) indicated that SHR168442 gave excellent efficacy in both preclinical mouse psoriasis models with favorable PK profile and significant suppression of signature cytokines, IL-17, TNFα and IL-6.

This novel RORγ antagonist may represent a new option as topical targeted therapeutics for mild to moderate psoriasis patients. Results from further clinical evaluation of this specific mechanism for the treatment of mild to moderate psoriasis are highly anticipated. Furthermore, Th17 pathway is associated with many other autoimmune diseases, such as asthma and severe ocular surface autoimmunity^33,34^. These autoimmune diseases are accessible locally, for example, topical cream, inhaler, or eye drops, and so on. The approach described in this study that RORγ antagonists with high exposure in target tissues and low in systemic circulation may be suitable for the treatment of other inflammatory disorders that are accessible locally.

## Methods

### Molecular docking

Molecular modeling studies were done using a laptop computer with an Intel i7-7700HQ, 2.8 GHz CPU and 16 GB RAM running the Microsoft Windows 10 professional operating system. The software package Molecular Operating Environment (MOE), Chemical Computing Group Inc., Montreal, H3A2R7 Canada, htpp://www.chemcomp.com, was used for the modeling study. PDB structures were downloaded and prepared using Structure Preparation within MOE, including adjusting hydrogens and lone pairs using Protonate3D^35^. Sequence and structures alignments were performed using MOE Align/Superpose function. The crystal structure of RORγ LBD in complex with small molecule antagonist (PDB accession code: 4NIE) were downloaded from Protein Data Bank (PDB)^36^. The co-crystal ligand was modified to GSK805. By selecting GSK805 and the surrounding residues within 4.5 Å, iterative energy minimizations with gradually increased tether deviations were performed to relax the clash between the ligand and protein while forming multiple interactions between ligand and protein. Finally poses were evaluated by using GBVI/WDA dG score and ligand strain energy with the rigid receptor and AMBER10: EHT force field with R-Field solvation. Compounds 1–3 were modeled using the similar procedure as that for GSK805.

### LanthaScreen TR-FRET human RORγ coactivator assay

The assay was performed with GST-tag RORγ LBD (25 ng/reaction, Creative Biomart), 0.6 μM Fluorescein-D22 coactivator peptide and 8 nM Tb anti-GST antibody (Invitrogen) at room temperature for 60 minutes. The fluorescent signals were measured with 520 nm emission and 495 nm excitation on Tecan Infinite. The data were calculated using DMSO control as 0% inhibition and non-protein control as 100% inhibition. IC_50_ values were calculated using non-linear regression analysis of GraphPad Prism in all assays.

### Human IL-17 production assay using human PBMC

Human PBMCs (Zenbio) in TexMACS media were stimulated by cytostim (Miltenyi) for 72 h following the vendor's instruction. Human IL-17A in cell culture supernatant was measured using AlphaLISA human IL-17A Biotin-free detection kit (Perkin Elmer) according to the manufacturer’s protocol.

### Human RORγ and RORα reporter assays

Cell-based luciferase reporter assays for RORα and RORγ (Indigo Biosciences) were performed according to the manufacturer’s protocol.

### Mouse IL-17 production assay using mouse splenocyte

Mouse splenocytes (Zenbio) were stimulated by Mouse Th17 Differentiation Media (R&D Systems) according to the manufacturer’s protocol. On day 5, compounds were added and then incubated for 72 h, and IL-17 in the supernatant was measured using IL-17A homodimer (mouse) AlphaLISA detection kit (Perkin Elmer) according to the manufacturer’s protocol.

### In vitro assessment of microsomal stability

The compound was mixed with 0.5 mg/ml human liver microsome (Thermal Fisher) at a final concentration of 1 µM in 100 mM potassium phosphate buffer, pH 7.4. The reactions were initiated by the addition of 1 mM NADPH. Samples in duplicate were incubated at 37 °C with mixing. 100 µL aliquots were transferred after 0, 5, 10, 30, 45, 60 minutes to a new tube with 300 µL acetonitrile, incubated on ice for 30 minutes, centrifuged at 1800×*g* at 4 °C for 10 minutes. The supernatants were transferred to glass vials subjected to LC/MS analysis. Peak areas were measured to calculate half-life according to the formula t_1__/2_ = − 0.693/slope of linear regression analysis of ln [concentration] versus incubation time. Similar methods were applied to access mouse and rat liver microsomal stability of the compounds.

### Dermal PK analyses of mice, rats, and miniature pigs

The dermal PK studies of Compound 1 in Vaseline were performed in dorsal skin of female C57BL/6 and male BALB/c mice via topical route. The skin (1.5 cm × 2 cm) was shaved one day prior to the dosing. At tissue collecting time point, the skin surface was decontaminated (twice with hand soap, twice with water, alternatively, and dried with tissue swabs). Stratum corneum is removed with tape strips for five times. The tissues were homogenized for PK analyses of Compound 1 by LC/MS.

Similar procedures were carried out for dermal PK of female SD rats and female Bama miniature pigs. Briefly, each area of topical application was 4 cm × 9 cm for SD rats and 6 cm × 6 cm for miniature pigs. After decontamination, the biopsy sections of SD rats were tape stripped for five times and those of miniature pig for twenty-five times, respectively. Blood and dorsal skin tissues of the animals were collected and analyzed for the compounds via LC/MS. The experimental protocol used in this study was approved by the Institutional Animal Care and Use Committees (IACUC). The experiments were conducted in accordance with the Guiding Principles for the Care and Use of Laboratory Animals and complied with the ARRIVE guidelines.

### Oral PK analyses of compounds in SD rats

Compounds were dissolved in 5% DMSO, 5% tween 80 and then added 90% saline. Male SD rats were dosed with the compound via oral (2 mg/kg or 5 mg/kg) routes, respectively after fasting overnight. Blood of the animals were collected at indicated time post dosing. Blood samples were then prepared for plasma and analyzed for the compounds using validated LC/MS assays. The experimental protocol used in this study was approved by IACUC. The experiments were conducted in accordance with the Guiding Principles for the Care and Use of Laboratory Animals and complied with the ARRIVE guidelines.

### IMQ-induced psoriasis-like skin inflammation and topical treatment with SHR168442 in vaseline

Psoriasis was induced by topical application of IMQ cream on the shaved dorsal skin as described by Leslie van der Fits et al.^18^. SHR168442 in Vaseline (n = 10 per group) was treated once a day (QD) from 3 days prior to day 1, and then twice a day (BID) from day 1 to day 6. The animal was sacrificed at 3 h after the first dose in the morning of day 7. Erythema, scaling, and thickness of the back skin were scored independently daily on a scale from 0 to 4. Blood and back skin tissues from the study were collected at 3 h (n = 4) post dosing in the morning of day 6. The plasma and skin tissues after tape stripping were analyzed for Compound 1 as described above. The in-life portion of the study was carried out at Washington Biotechnology (Baltimore, MD). The housing conditions and in-life procedures were approved by IACUC. The experiments were conducted in accordance with the Guiding Principles for the Care and Use of Laboratory Animals and complied with the ARRIVE guidelines.

### IL-23-induced mouse psoriasis-like skin inflammation and topical treatment with SHR168442 in vaseline

Psoriasis was induced by intradermal injection of IL-23 on mouse skin as described by Rizzo et al*.*^19^. Three days later after shaving (day 1), two injections of IL-23 (0.5 µg protein per mouse) were performed on both sides of the dorsal skin (except naïve group) for 8 days. SHR168442 in Vaseline was topically applied daily starting three day before IL-23 injections, and then twice a day starting on the day of IL-23 injections. The mice were weighed daily, and the skin were scored daily on a scale from 0 to 4. At day 8, the skin thickness was measured in live mice with a caliper and the animal was sacrificed 3 h after the first topical application. The in-life portion of the study was carried out at Nanjing University (Nanjing, China). The experimental protocol used in this study was approved by IACUC in Nanjing, China. The experiments were conducted in accordance with the Guiding Principles for the Care and Use of Laboratory Animals and complied with the ARRIVE guidelines.

### Cytokine measurements

IL-17A, IL-6 and TNFα were determined in plasma collected at day 8 using a cytometric bead array (BD Biosciences) following the vendor's instruction.

### Statistical analysis

The significance of differences between groups was determined by one-way ANOVA (Prism GraphPad Software). The significance of differences between groups was determined by one-way ANOVA, *p < 0.05, **p < 0.01, ***p < 0.001, ****p < 0.0001.

### Preparation of SHR168442

The chemical structure of SHR168442 is ((S)-3-(4,6-dichloro-5-(4,4-difluoropiperidin-1-yl)-1H-benzo[d]imidazol-2-yl)-3-(4-(ethylsulfonyl) phenyl) propan-1-ol). It was prepared in Eternity Bioscience Inc. according to the following procedure. 3,5-Dichloro-4-(4,4-difluoropiperidin-1-yl)benzene-1,2-diamine and 4-ethoxy-2-(4-(ethylsulfonyl)phenyl)-4-oxobutanoic acid was coupled with 1-ethyl-3-(3-dimethylaminopropyl)carbodiimide hydrochloride (EDCl) and Hydroxybenzotriazole in dimethylformamide (DMF) to get a mixture of N-(2-amino-3,5-dichloro-4-(4,4-difluoropiperidin-1-yl)phenyl)-2-(4-(ethylsulfonyl)phenyl)acetamide and N-(6-amino-2,4-dichloro-3-(4,4-difluoropiperidin-1-yl)phenyl)-2-(4-(ethylsulfonyl)phenyl)acetamide. MS (ESI): m/z = 592 (M + H)^+^. The mixture of N-(2-amino-3,5-dichloro-4-(4,4-difluoropiperidin-1-yl)phenyl)-2-(4-(ethylsulfonyl)phenyl)acetamide and N-(6-amino-2,4-dichloro-3-(4,4- difluoropiperidin-1-yl)phenyl)-2-(4-(ethylsulfonyl)phenyl)acetamide was treated with glacial acetic acid at 80 °C for 2 h to afford 2-(4-(ethylsulfonyl)benzyl)-4,6-dichloro-5-(4,4-difluoropiperidin-1-yl)-1H-benzo[d]imidazole. MS (ESI): m/z = 573 (M + H)^+^. 2-(4-(Ethylsulfonyl)benzyl)-4,6-dichloro-5-(4,4- difluoropiperidin-1-yl)-1H-benzo[d]imidazole was reduced to the alcohol by LiAlH4 at 0 °C in tetrahydrofuran (THF) to get the racemic product 3-(4,6-dichloro-5-(4,4-difluoropiperidin-1-yl)-1H-benzo[d]imidazol-2-yl)-3-(4-(ethylsulfonyl)phenyl)propan-1-ol. The racemic mixture was separated to the “S” (SHR168442) and “R” isomers by chiral HPLC according to the following method.

Separation conditions: CHIRALCEL OZ-H(OZH00CD-VC005, 0.46 cm I.D. × 15 cm L; mobile phase : Hexane/EtOH/DEA = 70/30/0.1(V/V/V); flow rate: 1.0 mL/min), The corresponding fractions were collected and concentrated under reduced pressure to obtain the title compounds. The “S” isomer chiral HPLC analysis: retention time 7.640 min, chiral purity: 100% (chromatographic column: OD Phenomenex Lux Cellulose-1 150 × 4.6 mm, 5 µm; mobile phase: ethanol/hexane = 15:85 (v/v). 1H NMR (CD_3_Cl, 400 MHz): 7.90 (d, 8.0 Hz, 2H), 7.72 (s, 1H), 7.68 (d, 8.0 Hz, 2H), 4.68 (t, 8.0 Hz, 1H), 3.76 (t, 8.0 Hz, 2H), 3.71(m, 4H), 3.14–3.12 (m, 2H), 2.65–2.60 (m, 1H), 2.38–2.35 (m, 1H), 2.10–2.10 (m, 4H), 1.23–1.19 (t, 8.0 Hz, 3 H). 13C NMR (DMSO-d6, 400 MHz): 158.9527, 158.3818, 148.0020, 141.7011, 140.8159, 138.7433, 137.5245, 132.8604, 129.3228, 124.2036, 118.5185, 111.1078, 58.5615, 49.6160, 47.1413, 41.8436, 37.5654, 35.2187, 7.5737. HRMS: 532.1036 (M + H)^+^.

## Supplementary information

Supplementary information.

## Data Availability

All data needed to evaluate the conclusions in the paper are presented in the paper and the supplementary information.
