# Supplementary material for: Discovery of a novel RORγ antagonist with skin-restricted exposure for topical treatment of mild to moderate psoriasis
Source: Sci Rep. 2021 Apr 28;11:9132. doi: 10.1038/s41598-021-88492-1 (PMC8080595; doi:10.1038/s41598-021-88492-1)
Supplement: Supplementary file 1 — Supplementary information. [file 41598_2021_88492_MOESM1_ESM.docx]

**Discovery of a novel RORγ antagonist with skin-restricted exposure for topical treatment of mild to moderate psoriasis**

Suxing Liu^1⁺^*, Dong Liu^1⁺^, Ru Shen^1^, Di Li^1^, Qiyue Hu^2^, Yinfa Yan^1^, Jiakang Sun^2^, Fengqi Zhang^1^, Hong Wan^2^, Ping Dong^2^, Jun Feng^2^, Rumin Zhang^1^, Jing Li^1^, Lianshan Zhang^2^, Weikang Tao^2^

^1^Eternity Bioscience Inc. 6 Cedarbrook Drive, Cranbury, NJ 08512, USA

^2^Shanghai Hengrui Pharmaceutical Co. Ltd. 279 Wenjing Road, Shanghai 200245, China

*Corresponding author: Suxing Liu, E-mail: [lius@eternitybioscience.com](mailto:lius@eternitybioscience.com)

⁺these authors contributed equally

# Supplementary Methods

# Preparation of compound 1, 4,6-dichloro-2-(2-chloro-4-(ethylsulfonyl) benzyl)-5-(2-(trifluoromethoxy)phenyl)-1H-benzo[d]imidazole

Step 1. Preparation of methyl 2-(2-fluoro-4-nitrophenyl) acetate

To a solution of 2-(2-chloro-4-nitrophenyl)acetic acid (4.3 g, 0.02 mol) in methanol (20 mL) was added concentrated sulfuric acid (1 mL). The solution was heated to reflux overnight. After cooling, the mixture was concentrated to a small amount and partitioned between ether (30 mL) and water. The ether phase was separated and washed with saturated sodium bicarbonate solution (30 mL). The organic phase was then separated again, washed with water, and dried over MgSO_4_. The solid was filtered off and evaporated the solvent to give the product pure enough for the next step (4.0 g, 88%), MS (ESI): m/z = 230 (M+H)^+^.

Step 2. Preparation of 2-(2-fluoro-4-aminophenyl) acetic acid methyl ester


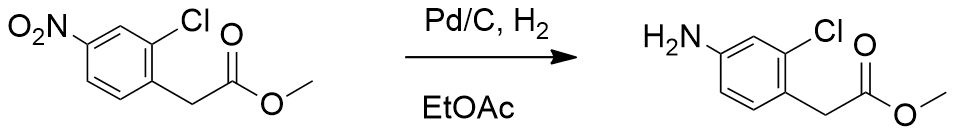


To a 50 mL round bottom flask, were added 2-(2-chloro-4-nitrophenyl) acetic acid methyl ester (4.0 g, 0.018 mol) and ethyl acetate (30 mL). To the same flask, 10% palladium on activated carbon (0.5 g) was added. The reaction mixture was stirred under hydrogen gas atmosphere for 5 hours. The reaction mixture was filtered through a pad of celite. The filtrate was evaporated under reduced pressure to get the title compound (3.0 g, 86%), MS (ESI): m/z = 200 (M+H)^+^.

Step 3. Preparation of 2-(2-chloro-4-mercaptophenyl) acetic acid

A solution of sodium nitrite (1.06 g, 0.015 mol) in 16 mL of water was added dropwise at 0°C, to a stirred suspension of 2-(4-amino-2-fluorophenyl) acetic acid methyl ester (2.8 g, 0.015 mol) in 50 mL of water and 3.8 mL of concentrated hydrochloric acid. After the addition was complete, the reaction mixture was stirred at the same temperature for additional 60 minutes. This cold diazonium salt solution was then added dropwise at room temperature to a mixture of potassium O-ethyl carbonodithioate (2.8 g), 50 mL of water and 16 mL of 2 M sodium carbonate solution and was heated to 45°C until gas evolution stopped. The mixture was cooled to room temperature, and acidified to pH = 1 with concentrated hydrochloric acid. The xanthogenate ester was extracted with ether. Solvent was evaporated to give a dark red liquid ethyl 2-(2-chloro-4-mercaptophenyl) acetate (3.5 g), MS (ESI): m/z = 231 (M+H)^+^.

The above oily product was dissolved in ethanol (10 mL), a solution of KOH (1.8 g) in water (10 mL) was added and the mixture heated to reflux overnight. The mixture was concentrated to a small amount and acidified with concentrated HCl. The product was extracted with ethyl acetate (10 mL × 3). The combined organic phase was dried over MgSO_4_, and then the solid was filtered off. Evaporation of the solvent to afford the crude product (2.5 g, 89%), MS (ESI): m/z = 203 (M+H)^+^.

Step 4. Preparation of ethyl 2-(4-(ethylthio)-2-chlorophenyl) acetate

2-(2-chloro-4-mercaptophenyl) acetic acid (2.5 g, 0.013 mol) was dissolved in dimethylformamide (DMF) (25 mL), followed by the addition of cesium carbonate (13.0 g, 0.039 mol). The mixture was stirred for 10 minutes before iodoethane (6.0 g, 0.039 mol) was added. The mixture was stirred at room temperature overnight. The reaction mixture was partitioned between ethyl acetate (20 mL) and water (30 mL). The organic phase was separated and dried over MgSO_4_. The solid was filtered off and the solvent was evaporated to dryness. This product was purified by flash chromatography with hexane/ethyl acetate to afford an oil (2.8 g, 86%), MS (ESI): m/z = 259 (M+H)^+^.

Step 5. Preparation of ethyl 2-(4-(ethylsulfonyl)-2-chlorophenyl) acetate

Ethyl 2-(4-(ethylthio)-2-chlorophenyl) acetate (2.8 g, 0.012 mol) was dissolved in dichloromethane (DCM) (50 mL). The solution was cooled to 0°C with an ice bath. MCPBA (6.0 g) was added in portions. The reaction mixture was stirred at room temperature overnight, and then filtered to remove the solid. The filtrate was washed with saturated sodium carbonate solution (30 mL × 2), water (30 mL), brine (30 mL), and then dried over magnesium sulfate, and concentrated. The residue was purified by column chromatography with hexane/ethyl acetate to afford the target compound ethyl 2-(4-(ethylsulfonyl)-2-fluorophenyl) acetate (2.0 g, 64%), MS (ESI): m/z = 291 (M+H)^+^.

Step 6. Preparation of 2-(4-(ethylsulfonyl)-2-chlorophenyl) acetic acid

A solution of ethyl 2-(4-(ethylsulfonyl)-2-chlorophenyl)acetate (2.0 g, 7.3 mmol) in ethanol (30 mL) was mixed to a solution of NaOH (1.0 g) in water (10 mL). The reaction mixture was stirred at room temperature overnight. Ethanol was removed under reduced pressure, and 20 mL of water was added. The aqueous phase was acidified to pH = 1 with 6 M HC1, and then extracted with ethyl acetate (50 mL × 3). The combined organic phases were washed with brine (50 mL), dried over magnesium sulfate, and concentrated to afford an oil which solidified upon standing (1.6 g, 90%), MS (ESI): m/z = 263 (M+H)^+^.

Step 7. Preparation of N-(6-amino-3-bromo-2,4-dichlorophenyl)-2-(4-(ethylsulfonyl)-2-fluorophenyl) acetamide

1-Ethyl-(3-(3-dimethylamino) propyl)-carbodiimide hydrochloride (1.0 g, 5 mmol) and benzotriazol-1-ol (0.7 g, 5 mmol) were added into a cooled solution (ice water bath) of 2-bromo-1,3-dichloro-4,5-diamino-benzene (1.28 g, 5 mmol) and 2-(4-(ethylsulfonyl)-2-chlorophenyl)acetic acid (1.23 g, 5 mmol) in DMF (10 mL) portion wise. After the addition was completed, the mixture was stirred for 60 minutes, allowed to warm-up to room temp, and stirred overnight. The mixture was partitioned between water (50 mL) and ethyl acetate (50 mL). The organic phase was separated and dried over MgSO_4_ and filtered. The solvent was evaporated under reduced pressure to leave an off-white solid, which was further purified by flash chromatography with hexane/ethyl acetate to afford the product as a solid (1.7 g, 70%), MS (ESI): m/z = 501 (M+H)^+^.

Step 8. Preparation of 5-bromo-4,6-dichloro-2-(4-(ethylsulfonyl)-2-chlorobenzyl)-1H-benzo[d]imidazole

N-(6-amino-3-bromo-2,4-dichlorophenyl)-2-(4-(ethylsulfonyl)-2-chlorophenyl) acetamide (step 7) (1.7 g, 3.5 mmol) was mixed with acetic acid (10 mL). The mixture was heated to 100°C for 4 hours, and then cooled. The solvent was evaporated under reduce pressure and the residue was dissolved in dichloromethane and washed with saturated sodium bicarbonate, dried over MgSO_4_. This product was purified by flash chromatography with hexane/ethyl acetate to afford the product as a white solid (1.2 g, 73%), MS (ESI): m/z = 482 (M+H)^+^.

Step 9. Preparation of 4,6-dichloro-2-(4-(ethylsulfonyl)-2-chlorobenzyl)-5-(2-(trifluoromethoxy) phenyl)-1H-benzo[d]imidazole

A mixture of 5-bromo-4,6-dichloro-2-(4-(ethylsulfonyl)-2-chlorobenzyl)-1H-benzo[d]imidazole

(Step 8) (480 mg, 1 mmol), (2-(trifluoromethoxy) phenyl) boronic acid (615 mg, 3 mmol), tris-(dibenzylideneacetone) dipalladium (0) (60 mg), tri(tert-butyl) phosphonium tetrafluoroboronate (60 mg) and sodium carbonate (2 M solution) in 1,4-dioxane (3 mL) was degassed, sealed and heated to 100°C under microwave irradiation for 1 hour. The volatile solvents were removed under reduced pressure. The residue was directly loaded onto a ISCO solid cartridge and flashed with hexane/ethyl acetate to afford the product as a white solid (393 mg, 70% yield), ^1^H NMR (CDCl_3_, 400 mHz): 13.26 (s, 0.7 H), 12.98 (s, 0.3 H), 7.99 (s, 1 H), 7.80 (t, 1 H), 7.70-7.78 (m, 2 H), 7.60 (t, 1 H), 7.50-7.55 (m, 2H), 7.30-7.45 (m, 1 H), 4.50 (s, 2 H), 3.30-3.40 (m,, 2 H), 1.10-1.20 (t, 3 H). ^13^C NMR (DMSO-d6, 400 mHz): 135.2833, 134.8238, 134.7962, 133.2602, 133.1527, 132.2388, 131.1287, 130.9445, 130.6217, 130.2296, 128.8755, 128.7651, 127.9266, 127.8606, 127.4273, 127.3237, 127.1868, 127.0362, 126.8519, 123,7163, 121.4510, 121.0995, 121.0071, 119.4052, 118.1753, 116.4348, 111.2076, 49.4370, 33.2880, 33.0431, 7.4755, 0.5735. HRMS: 564.9937 (M+H)^+^.

# Preparation of compound 2, 4,6-dichloro-5-(4,4-difluoropiperidin-1-yl)-2-(4-(ethylsulfonyl) benzyl)-1H-benzo[d]imidazole

Step1. Preparation of 2,4-dichloro-3-(4,4-difluoropiperidin-1-yl)-6-nitroaniline

The mixture of 2,3,4-trichloro-6-nitroaniline (241 mg, 1 mmol), 4,4-difluoropiperidine (190 mg, 1.2 mmol) and DIEA (390 mg, 3 mmol) in 8 mL DMSO was heated in a sealed vessel to 108°C overnight. After cooling, the reaction mixture was partitioned between water (30 mL) and ethyl acetate (10 mL). The organic phase was separated and dried over MgSO_4_. The solid was filtered off, the solvent was evaporated, and the residue was directly flashed with hexane/ethyl acetate to afford a yellow solid 280 mg (yield 85%), MS (ESI): m/z = 326 (M+H) ^+^.

Step 2. Preparation of 3,5-dichloro-4-(4,4-difluoropiperidin-1-yl) benzene-1,2-diamine

The mixture of 2,4-dichloro-3-(4,4-difluoropiperidin-1-yl)-6-nitroaniline (200 mg, 0.68 mmol), and Pd/C (20 mg) in 15 mL methanol was hydrogenated with a hydrogen balloon for 2 hours. The catalyst was filtered off and the solvent was evaporated to leave tan residue that was directly flashed with hexane/ethyl acetate to afford an oil product 120 mg (yield 66%), MS (ESI): m/z = 296 (M+H) ^+^.

# Step 3. Preparation of 4,6-dichloro-5-(4,4-difluoropiperidin-1-yl)-2-(4-(ethylsulfonyl) benzyl)-1H-benzo[d]imidazole

1-Ethyl-(3-(3-dimethylamino) propyl)-carbodiimide hydrochloride (20 mg, 0.1 mmol) and hydroxybenzotriazol-1-ol (13.5 mg, 0.1 mmol) were added into a cooled solution (ice water bath) of 3,5-dichloro-4-(4,4-difluoropiperidin-1-yl) benzene-1,2-diamine (29.6 mg, 0.1 mmol) and 2-(4-(ethylsulfonyl)phenyl)acetic acid (22.8 mg, 0.01 mmol) in DMF (1 mL). After the addition was completed, the mixture was stirred and allowed to warm-up to room temperature and stirred overnight. The mixture was partitioned between water (50 mL) and ethyl acetate (50 mL). The organic phase was separated and dried over MgSO_4_, and then filtered. The solvent was evaporated under reduced pressure to leave an off-white solid, which was mixed with acetic acid (1 mL). The mixture was heated to 80°C for 2 hours, and then cooled. The solvent was evaporated under reduce pressure and the residue was dissolved in ethyl acetate, washed with saturated sodium bicarbonate, and then dried over MgSO_4_. This product was purified by flash chromatography with hexane/ethyl acetate to afford the product as a white solid 26 mg (60%), ^1^H NMR (CDCl_3_, 400 mHz): 7.89 (d, 7.88 Hz, 2 H), 7.72 (s, 1 H), 7.55 (d, 7.88 Hz, 2 H), 4.37 (s, 2 H), 3.36 (m, 4 H), 3.13 (t, 7.24 Hz, 2 H), 2.14 (m, 4 H), 1.30 (t, 7.24 Hz, 3 H); ^13^C NMR (DMSO-d6, 400 mHz): 153.9099, 141.8400, 136.5220, 135.2138, 128.1074, 127.2358, 126.5416, 126.4308, 126.2375, 123.6507, 112.0182, 53.2446, 47.5405, 33.3162, 33.1010, 32.9257, 5.4700. MS (ESI): m/z = 488 (M+H)^+^.

**Supplementary Table S1. The screening results of SHR168442 in SAFETYscan47 panel**

| **Assay Name** | **Assay Target** | **Mode** | **% Response** | | |
| --- | --- | --- | --- | --- | --- |
|  |  |  | **Replicate 1** | **Replicate 2** | **Average** |
| Calcium Flux | ADORA2A | Agonist | 0.0 | 14.7 | 7.3 |
|  |  | Antagonist | 5.4 | 24.9 | 15.1 |
|  | ADRA1A | Agonist | 0.0 | 0.0 | 0.0 |
|  |  | Antagonist | 0.0 | 0.0 | 0.0 |
| cAMP | ADRA2A | Agonist | 0.0 | 0.0 | 0.0 |
|  |  | Antagonist | 21.2 | 16.4 | 18.8 |
|  | ADRB1 | Agonist | 0.0 | 0.0 | 0.0 |
|  |  | Antagonist | 20.0 | 12.1 | 16.1 |
|  | ADRB2 | Agonist | 0.0 | 0.0 | 0.0 |
|  |  | Antagonist | 21.3 | 23.3 | 22.3 |
| Calcium Flux | AVPR1A | Agonist | 4.5 | 0.0 | 2.3 |
|  |  | Antagonist | 0.0 | 9.5 | 4.7 |
|  | CCKAR | Agonist | 0.0 | 0.0 | 0.0 |
|  |  | Antagonist | 0.0 | 0.0 | 0.0 |
|  | CHRM1 | Agonist | 0.0 | 0.0 | 0.0 |
|  |  | Antagonist | 3.2 | 0.0 | 1.6 |
| cAMP | CHRM2 | Agonist | 0.0 | 0.0 | 0.0 |
|  |  | Antagonist | 17.5 | 32.9 | 25.2 |
| Calcium Flux | CHRM3 | Agonist | 0.0 | 0.0 | 0.0 |
|  |  | Antagonist | 0.0 | 4.6 | 2.3 |
| cAMP | CNR1 | Agonist | 0.0 | 0.0 | 0.0 |
|  |  | Antagonist | 3.1 | 3.8 | 3.5 |
|  | CNR2 | Agonist | 0.0 | 0.0 | 0.0 |
|  |  | Antagonist | 0.0 | 0.0 | 0.0 |
|  | DRD1 | Agonist | 0.0 | 0.0 | 0.0 |
|  |  | Antagonist | 27.2 | 25.2 | 26.2 |
|  | DRD2S | Agonist | 0.0 | 0.0 | 0.0 |
|  |  | Antagonist | 12.3 | 13.2 | 12.7 |
| Calcium Flux | EDNRA | Agonist | 0.0 | 0.1 | 0.1 |
|  |  | Antagonist | 0.0 | 0.0 | 0.0 |
|  | HRH1 | Agonist | 2.1 | 0.7 | 1.4 |
|  |  | Antagonist | 0.0 | 0.0 | 0.0 |
| cAMP | HRH2 | Agonist | 0.0 | 0.5 | 0.2 |
|  |  | Antagonist | 23.2 | 17.8 | 20.5 |
|  | HTR1A | Agonist | 1.8 | 0.8 | 1.3 |
|  |  | Antagonist | 35.3 | 20.2 | 27.7 |
|  | HTR1B | Agonist | 0.0 | 0.0 | 0.0 |
|  |  | Antagonist | 15 | 13.3 | 14.2 |
| Calcium Flux | HTR2A | Agonist | 0.0 | 0.0 | 0.0 |
|  |  | Antagonist | 0.0 | 6.0 | 3.0 |
|  | HTR2B | Agonist | 0.0 | 0.0 | 0.0 |
|  |  | Antagonist | 2.6 | 15.9 | 9.3 |
| cAMP | OPRD1 | Agonist | 0.0 | 0.0 | 0.0 |
|  |  | Antagonist | 21.9 | 9.6 | 15.7 |
|  | OPRK1 | Agonist | 0.0 | 0.0 | 0.0 |
|  |  | Antagonist | 0.0 | 0.0 | 0.0 |
|  | OPRM1 | Agonist | 0.0 | 0.0 | 0.0 |
|  |  | Antagonist | 38.7 | 48.5 | 43.6 |
| NHR Nuclear Translocation | AR | Agonist | 0.0 | 0.0 | 0.0 |
|  |  | Antagonist | 0.0 | 0.0 | 0.0 |
| NHR Protein Interaction | GR | Agonist | 1.2 | 1.1 | 1.2 |
|  |  | Antagonist | 0.0 | 0.0 | 0.0 |
| Transporter | DAT | Blocker | 0.0 | 0.0 | 0.0 |
|  | NET | Blocker | 0.0 | 0.0 | 0.0 |
|  | SERT | Blocker | 0.0 | 0.0 | 0.0 |
| Ion Channel | CAV1.2 | Blocker | 0.0 | 0.0 | 0.0 |
|  | GABAA | Opener | 3.2 | 1.9 | 2.5 |
|  |  | Blocker | 4.6 | 10.1 | 7.4 |
|  | hERG | Blocker | 6.5 | 3.2 | 4.9 |
|  | HTR3A | Opener | 4.2 | 3.3 | 3.7 |
|  |  | Blocker | 7.2 | 0 | 3.6 |
|  | KvLQT1/minK | Opener | 5.7 | 8.3 | 7 |
|  |  | Blocker | 3.4 | 2.4 | 2.9 |
|  | nAChR(a4/b2) | Opener | 0.0 | 4.3 | 2.2 |
|  |  | Blocker | 0.0 | 0.0 | 0.0 |
|  | NAV1.5 | Blocker | 12.6 | 19.8 | 16.2 |
|  | NMDAR (1A/2B) | Opener | 5.7 | 8.3 | 7 |
|  |  | Blocker | 3.4 | 2.4 | 2.9 |
| Non-Kinase Enzymatic | AChE | Inhibitor | 0.4 | 0.4 | 0.4 |
|  | COX1 | Inhibitor | 0.0 | 0.0 | 0.0 |
|  | COX2 | Inhibitor | 0.0 | 0.0 | 0.0 |
|  | MAOA | Inhibitor | 10.6 | 8.9 | 9.8 |
|  | PDE3A | Inhibitor | 4.0 | 0.0 | 2.0 |
|  | PDE4D2 | Inhibitor | 0.0 | 0.0 | 0.0 |
| Kinase Binding | INSR | Inhibitor | 0.0 | 0.0 | 0.0 |
|  | LCK | Inhibitor | 8.4 | 17.2 | 12.8 |
|  | ROCK1 | Inhibitor | 5.6 | 8.9 | 7.2 |
|  | VEGFR2 | Inhibitor | 0.0 | 0.0 | 0.0 |
